# Supplementary material for: Developing Gold Nanoparticles-Conjugated Aflatoxin B1 Antifungal Strips
Source: Int J Mol Sci. 2019 Dec 12;20(24):6260. doi: 10.3390/ijms20246260 (PMC6941036; doi:10.3390/ijms20246260)
Supplement: Supplementary file 1 [file ijms-20-06260-s001.pdf]

International Journal of *Molecular Sciences*

MDPI

Supplementary Materials

1. Materials

Nitrocellulose membrane (HF075), Conjugate pad (SB-06), Sample pad (8965), Absorbent pad, Adhesive PVC backing 30 cm x 8 cm were purchased from (Millipore). The cutting machine was from Cutier (Shanghai, China). Biodot μAIRJET™ nanoliter aerosol Dispenser was XYZ3060 Series.

2. Preparation of Strips

The composition of the 8 cm strip, starting from the bottom, was as follows: the sample pad, the conjugate pad, the Nitrocellulose membrane and the absorbent pad.

3. Sample Pad

The cutting machine was cleaned with ethanol before use. The sample pads were cut into strips with a length of 1 cm and width of 3 mm. The sample pads were soaked in ethanol to disinfect and ddH2O for 20 minutes each. Subsequently, the sample pads were soaked into 0.01 M of Phosphate-buffered saline (PBS) (containing 0.5% Bovine serum albumin (BSA) and 0.1% Tween-20) at pH 7.4 for at least 4 hours at 4 °C, and incubated at 36 °C for 2 hours. The sample pads were dried at 37°C for 4 h and stored for 6 months at 4 °C.

4. Conjugate Pad Soak Method

The conjugate pads were cut into strips with a length of 2 cm and width of 3 mm. The conjugate pads were soaked in ethanol to disinfect and ddH2O for 20 minutes each. Consequently, the conjugate pads were soaked into 20 mL of conjugated AFB1 antibody + BSA + AuNPs OD 10 with 0.01M PBS containing 0.5% BSA, 6.5% sucrose, 2% trehalose and 0.05% triton at pH 7.4 for at least 4 hours at 4 °C, and incubated at 36 °C for 2 hours. The conjugate Pads were dried at 37 °C for 4 h and stored for 6 months at 4 °C.

5. Fabrication of Lateral-Flow Strip

Adhesive PVC backing card was 30 cm length and 8 cm width. The absorption pad was located at the top end of the PVC backing card. The Nitrocellulose (NC)membrane was located at the centre section of the PVC backing card, slightly overlapping the absorption pad . Goat anti-mouse IgG (0.5 mg/mL) was sprayed onto the NC membrane at a location close to the absorption pad at a volume of 1 μL/cm with a Biodot contact dispenser to form the control line (C line). The Aflatoxin B1 antigen (33 g/mL) was then sprayed onto the NC membrane alongside the control line nearest to the sample pad at a volume of 1 μL/cm to form the test line (T line). The Adhesive PVC backing card was cut into strips with a length of 8 cm and width of 3 mm. The prepared conjugated pads were attached to the other end of the PVC backing card, overlapping the NC membrane by 2 mm. The prepared sample pads were attached onto the conjugate pad overlapping by 2 mm . The cutting machine was cleaned thoroughly with ethanol before use, then lateral-flow strips were placed to be cut. The lateral-flow strips were manually assembled. The assembled strip cards were stored in a desiccator until further testing.

6. Sample Analysis

To perform the LFIAs , the prepared test strip was placed on a clean horizontal platform, 60 μL sample solution containing AFL was dropped onto the sample pad which migrated along the strip under capillary action. PBS without AFL was used as control and each sample test was repeated for 3 times under the same conditions. After appropriate immunoreaction time, the test strip was placed into Smartphone analysis reader, followed by recording the intensity of the test lines and control line to quantify the analytes.

7. Smartphone analysis

The smartphone was equipped with Central Processing Unit (CPU, MediaTek Helio X10 MT6795 processor) and Graphics Processing Unit (GPU, PowerVR G6200). The smartphone screen had a high resolution (1920 × 1080 p) and the screen pixel density was 441 ppi. The rear camera was based on complementary metal-oxide-semiconductor (CMOS) image sensor with 13 million pixels. The Java programming language was employed in the design of Android Studio 1.5 development environment and provided main functions of processing test strip images, analysis, and diagnosis in the smartphone. Black smartphone accessory (55 × 78 × 30 mm^3) and an ICTS cartridge (70 × 18 × 4.8 mm^3) were designed by SolidWorks software (Fig. S6). The improved Sobel operator was used in the software, which improved greatly the ability of distinguishing between the test area and background boundary information [20]. After the strip was inserted in exposure chamber, the image was taken by the camera under the LED light (365 nm, 1 W). The analysis period was 3 s. The imaging was carried out and transferred into a high-resolution black and white format. There, the signals of the T line and C line were represented by the black or grey and measured. The final number was then derived by the ratio of the T line intensity/C line intensity associated with every strip. The ratio of T-line density/C-line density and the concentrations of Alfatoxin B1 were treated with a 4-parameter logistic equation. (a = the minimum concentration, d = the maximum concentration, c = the point of inflection, b = Hill's slope of the curve.)

The equation:

$$Y = c\left(\frac{a-d}{X-d} - 1\right)^{\frac{1}{b}} \quad (1)$$

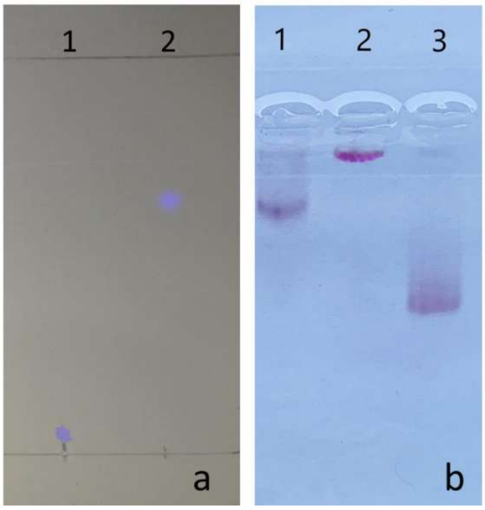

Figure S1. TLC and Gel electrophoresis of AuNPs. (**a**) TLC of AuNPs (1) Conjugated AuNPs (2) Aflatoxin B1 antibody. (**b**) Gel electrophoresis of (1) Plain, (2) Conjugated + antigen, (3) Conjugated AuNPs.

The Aflatoxin B1 were extracted from contaminated food samples (Peanut, bread, corn and rice).

A negative sample was observed on the LFIAs as visible purple lines at the C line and T line of the membrane. For a positive sample (AFB concentration is more than 10 ng/g), there was only one visible purple line at the control line observed on LFIAs (Figures S2–S5).

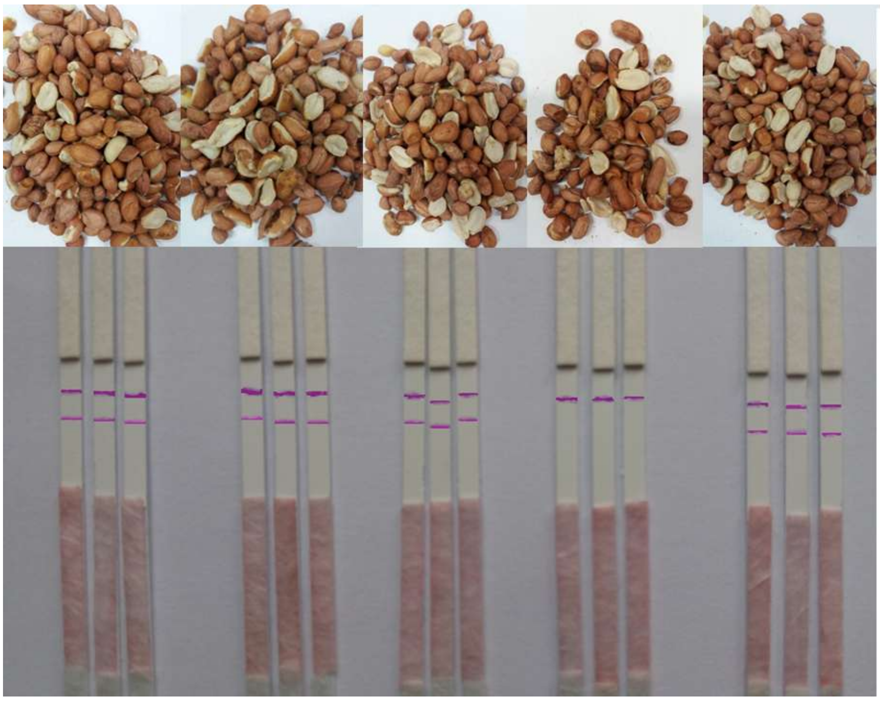

Figure S2. Aflatoxin conjugated AuNPs strip on peanut samples.

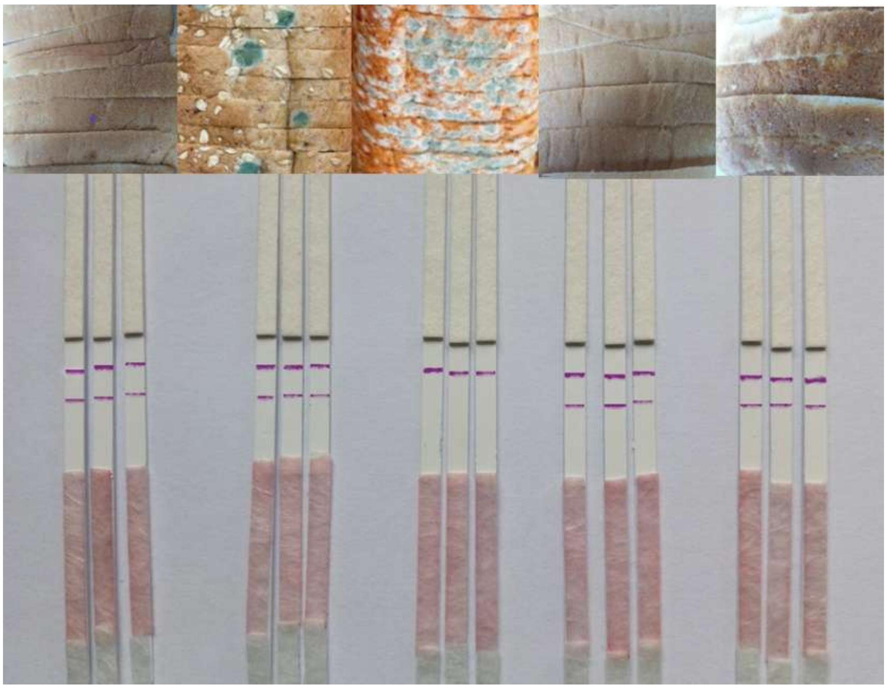

Figure S3. Aflatoxin conjugated AuNPs strip on bread samples.

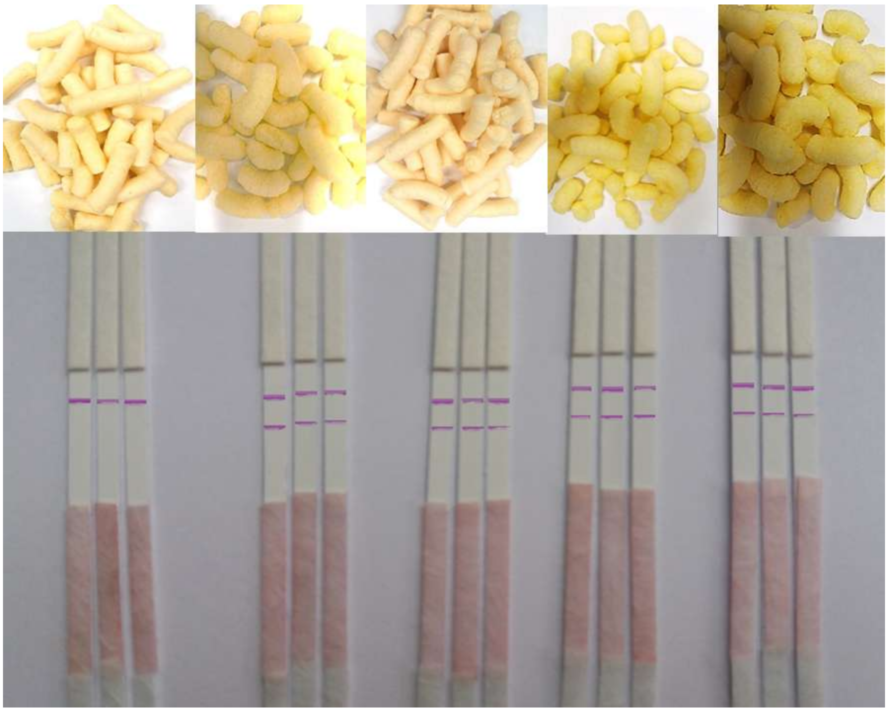

Figure S4. Aflatoxin conjugated AuNPs strip on corn snack samples.

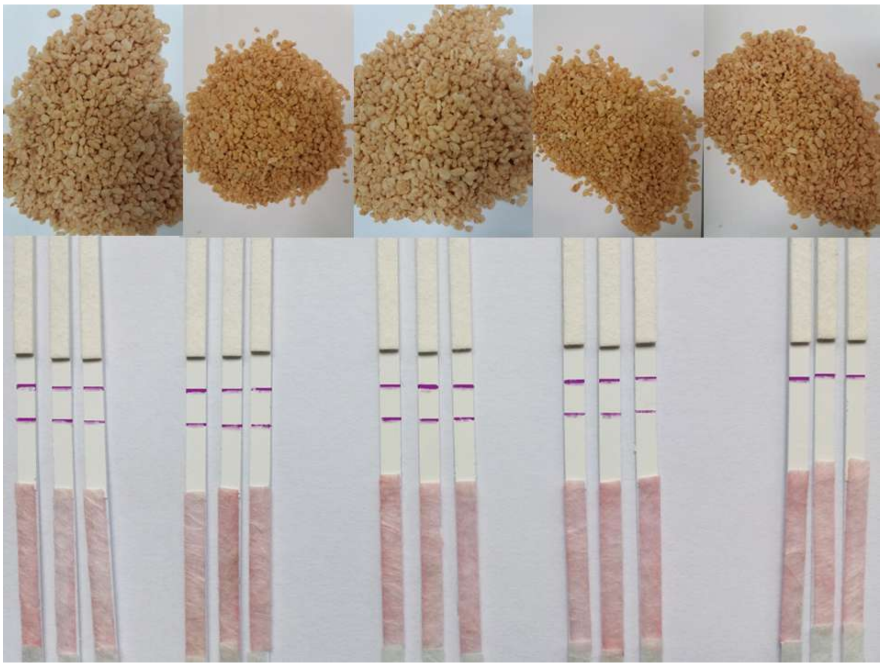

Figure S5. Aflatoxin conjugated AuNPs strip on rice snack samples.

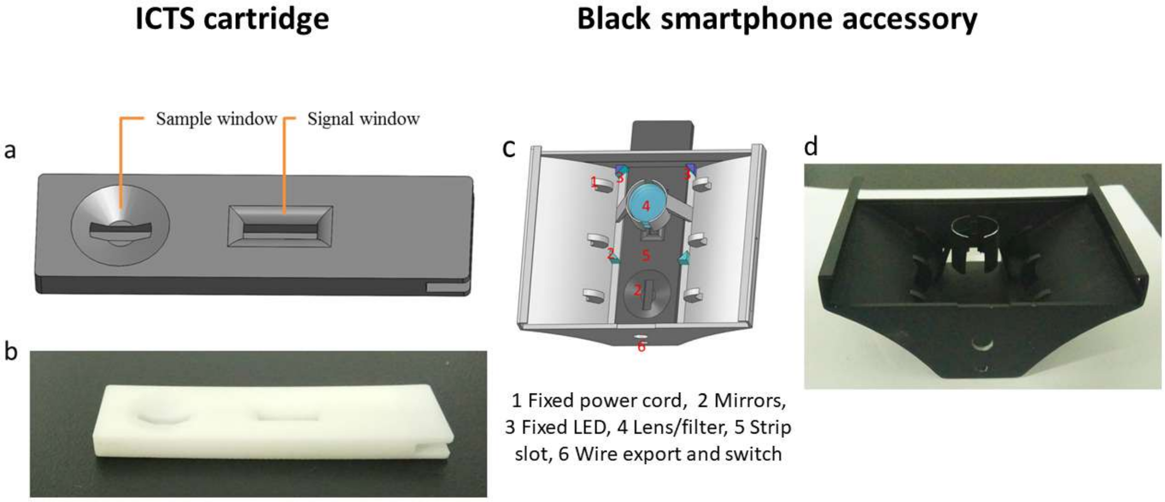

Figure S6. Images of Black smartphone accessory and ICTS cartridge.

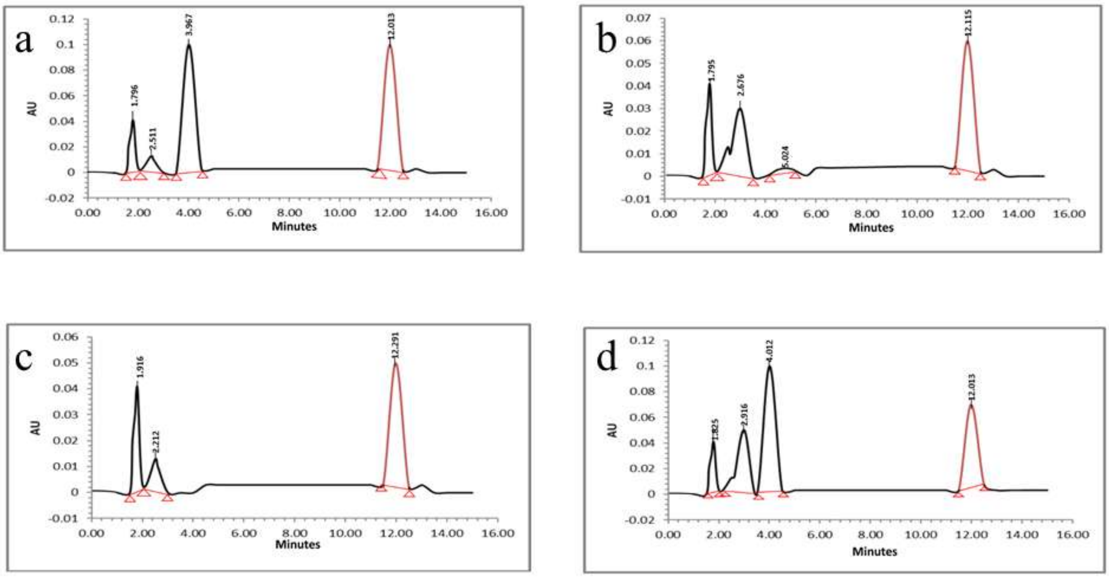

Figure S7. HPLC results showing AFB1 from the different food samples.
